# Is There Any Relationship Between Cervical Disc Herniation and Blood Inflammatory Response?

**DOI:** 10.7759/cureus.10161

**Published:** 2020-08-31

**Authors:** Kadri Burak Ethemoğlu, Yavuz Selim Erkoç

**Affiliations:** 1 Neurosurgery, Harran University, Şanlıurfa, TUR; 2 Neurosurgery, Kecioren Education and Research Hospital, Ankara, TUR

**Keywords:** disc herniation, inflammation, neutrophil-lymphocyte ratio, c - reactive protein

## Abstract

Objective

Inflammation plays an important role in the pathophysiology of disc herniation. The aim of this study was to evaluate blood neutrophil-lymphocyte ratio (NLR), platelet-lymphocyte ratio (PLR), and C-reactive protein (CRP) levels in cervical disc herniation (CDH) patients.

Materials and methods

We retrospectively analyzed the medical records of 126 patients with neck pain who were treated as inpatients at the Neurosurgery Department of Harran University Faculty of Medicine. The NLR, PLR, and CRP levels during hospital admissions were documented.

Results

The study included 73 patients with CDH and neck pain, 53 patients with normal cervical MR examination and neck pain, and 50 healthy control subjects. The group with cervical disc hernia had a significantly higher mean serum leucocyte count, neutrophil count, NLR, and CRP level compared to those with a normal MR but neck pain and the control group. NLR was significantly higher in the multi-level CDH group compared to the control group, while the single-level CDH and multi-level CDH had no significant difference with respect to mean serum inflammatory parameters.

Conclusion

Higher NLR and CRP in patients with CDH compared to patients with neck pain but normal cervical MR and the controls may be due to a developing inflammatory response. It may be speculated that among patients with neck pain, those with non-elevated NLR and CRP levels may have normal neck MR imaging, and in patients with elevated NLR and CRP levels, early protective approaches may play a preventive role in disc degeneration and cervical disc hernia development.

## Introduction

Neck pain is an important public health problem, and the lifetime prevalence rate of neck pain has been reported to be 48.5% [1,2]. Neck pain that occurs in the absence of underlying disease such as radiculopathy is also called mechanical neck pain [3]. The cervical intervertebral disc is a common source of neck pain [2]. It has been shown that pain occurs as a result of the release of local inflammatory cytokines in cervical disc herniation (CDH). Although these cytokines are reported as a part of pain cascade, it has also been reported that inflammation around disc herniation causes an increase in C-reactive protein (CRP) levels. In general, an increase in the plasma concentration of interleukin 6 (IL-6) produced by macrophages around the disc tissue causes an increase in CRP levels [4-6].

Recent studies have reported that increased neutrophil-lymphocyte ratio (NLR) and platelet-lymphocyte ratio (PLR) are novel inflammatory markers used to assess the prognosis of various disorders. NLR is a cheap and readily available inflammatory marker and is calculated by using neutrophil and lymphocyte levels obtained from full blood count [7]. It has been shown that blood NLR is a novel potential predictor of systemic inflammation in various disorders such as coronary heart disease, chronic diseases, and malignancy [8-10]. Similarly, a recent study has reported that a high NLR predicted glioblastoma multiforme in a statistically significant manner [11].

Similarly, it has been observed that increased PLR was associated with adverse prognosis among patients with stroke, malignancy, and aneurysmal subarachnoid hemorrhage [8,12,13]. Although the predictive values of increased NLR and PLR have been shown in a variety of disorders, the relationship of NLR, PLR, and CRP with CDH has never been studied. We aimed to study the differences in the systemic inflammatory response between patients with symptomatic disc herniation and healthy subjects.

## Materials and methods

All patients gave written informed consent prior to study entry, and the Harran University Faculty of Medicine local ethics committee approved the study protocol.

One hundred and seventy-six participants were included in the study; 126 patients who were under follow-up at the Department of Neurosurgery of Harran University Faculty of Medicine between February 2017 and December 2019 were enrolled in the study. The control group consisted of 50 healthy adults. The participants were divided into three groups. The first group consisted of patients with CDH and neck pain (n=73); the second group consisted of patients with neck pain but a normal cervical MR (n=53); and the third group consisted of healthy subjects (n=50). The group of patients with CDH was further categorized as single-level CDH (n=46) and multi-level CDH (n=27). The exclusion criteria for patients and healthy control groups included hematological, autoimmune, renal, and hepatic disorders, acute or chronic systemic infections, chronic diseases such as diabetes mellitus, hypertension, and inflammatory diseases, anti-inflammatory drug use, and malignancy.

The medical records of the patients were retrospectively reviewed. Age, sex, neurological examination findings, and cervical MRI findings were recorded. All patients’ blood parameters at admission were reviewed and mean serum leucocyte (u/mm^3^), neutrophil (K/μL), lymphocyte (K/μL), and platelet (u/mm^3^) count as well as CRP (mg/dl) levels were recorded. NLR was calculated by dividing neutrophil count by lymphocyte count; PLR was calculated by dividing platelet count by lymphocyte count.

Statistical analysis

Statistical analyses were performed using SPSS Statistics for Windows version 22.0 (IBM, Armonk, NY) software package. Descriptive statistics included mean ± standard deviation, number, and percentage. Normally distributed continuous variables were compared using Student’s t-test and non-normally distributed continuous variables using the Mann-Whitney U test. One-way analysis of variance (ANOVA) was used to compare more than two groups with normally distributed variables and the Kruskal-Wallis test was used for non-normally distributed continuous variables in more than two groups. Categorical variables were compared using Chi-square or Fisher’s test. Statistical significance was set at a p-value of <0.05.

## Results

A total of 176 records were reviewed. The study included 73 patients with CDH and neck pain (mean age: 42.52 ±10.41 years); 53 patients with normal cervical MR examination (mean age: 40.07 ±10.06 years) and neck pain; and 50 healthy control subjects (mean age 40.94 ±13.49 years). There was no significant difference between the groups with respect to sex distribution and mean age (Table 1). The group with cervical disc hernia had a significantly higher mean serum leukocyte count, neutrophil count, NLR, and CRP levels compared to those with a normal MR but neck pain and the control group. The study groups did not significantly differ with regard to mean serum lymphocyte count, platelet count, and PLR. Table 1 summarizes the demographic properties and laboratory findings of the patients.

**Table 1:** The demographic and laboratory characteristics of patients and control groups Group I consisted of patients with cervical disc herniation and neck pain; group II consisted of patients with neck pain but a normal cervical MR; group III consisted of healthy subjects; the data were presented as mean ±SD. P: significance between group I and group II; P1: significance between group I and group III; P2: significance between group II and group III; P3: within groups NLR: neutrophil-lymphocyte ratio; PLR: platelet–lymphocyte ratio; CRP: C-reactive protein; SD: standard deviation

Variable	Group I (n=73)		Group II (n=53)		Group III (n=50)		P		P1		P2		P3	
Age, years	42.52 ±10.41		40.07 ±10.06		42.52 ±13.49		0.105		0.271		0.903		0.08	
Male	42.5%		54.7%		57.4%		0.856		0.715		1		0.914	
Female	57.5%		45.3%		42,6%									
Leukocyte, u/mm^3^	9.39 ±2.76		7.66 ±1.70		7.86 ±1.75		0.000		0.001		0.658		0.000	
Neutrophils, K/μL	5.88 ±2.68		4.37 ±1.44		4.46 ±1.49		0.000		0.000		0.953		0.000	
Lymphocytes, K/μL	2.60 ±0.79		2.57 ±0.53		2.54 ±0.64		0.825		0.694		0.828		0.726	
NLR	2.55 ±2.30		1.76 ±0.73		1.85 ±0.78		0.000		0.009		0.644		0.001	
Platelet, u/mm^3^	2.87 ±78.06		296.04 ±63.84		308.63 ±76.43		0.639		0.166		0.661		0.178	
PLR	117.03 ±45.02		122.04 ±43.898		120.04 ±35.55		0.000		0.487		0.979		0.004	
CRP, mg/dL	0.74 ±1.13		0.31 ±0.77		0.48 ±1.09		0.000		0.048		0.001		0.000	

The categorization of the CDH group by the number of involved discs showed that there was no significant difference in the mean serum inflammatory parameters of the subgroups with single-level CDH and multi-level CDH, while NLR was significantly higher in the multi-level CDH group compared to the control group (Table 2).

**Table 2:** Comparisons of inflammatory markers between single-level and multi-level CDH subgroups The data were presented as mean ±SD. P: significance between single-level CDH and multi-level CDH; P1: significance between single-level CDH and control group; P2: significance between multi-level CDH and control group; P3: within groups CDH: cervical disc herniation; NLR: neutrophil-lymphocyte ratio; PLR: platelet-lymphocyte ratio; CRP: C-reactive protein; SD: standard deviation

Variable	Single-level CDH (n=46)	Multi-level CDH (n=27)	Control group (n=50)	P	P1	P2	P3
Leukocyte, u/mm^3^	9.39 ±2.76	7.66 ±1.70	7.86 ±1.75	0.458	0.007	0.003	0.003
Neutrophils, K/μL	5.88 ±2.68	4.37 ±1.44	4.46 ±1.49	0.727	0.002	0.006	0.002
Lymphocytes, K/μL	2.60 ±0.79	2.57 ±0.53	2.54 ±0.64	0.635	0.732	0.958	0.602
NLR	2.55 ±2.30	1.76 ±0.73	1.85 ±0.78	0.12	0.072	0.005	0.011
Platelet, u/mm^3^	287 ±78.06	296.04 ±63.84	308.63 ±76.43	0.15	0.736	0.330	0.784
PLR	117.03 ±45.02	122.04 ±43.898	120.04 ±35.55	0.515	0.797	0.286	0.602
CRP, mg/dL	0.29 ±0.60	0.32 ±0.87	0.48 ±1.09	0.852	0.151	0.069	0.139

## Discussion

Nerve root pain caused by disc herniation is attributed to both chemical and mechanical factors [14]. As a chemical effect, nucleus pulposus found in the inner part of a disc has been experimentally shown to induce an inflammatory-like reaction in the nerve root after the annulus fibrosus is torn [14,15]. It has been reported that pro-inflammatory substances including interleukins and cytokines like tumor necrosis factor-α (TNF-α) are expressed immunohistochemically in the region of disc herniation [16]. These cytokines increase the release of chemokine from the degenerated disc and the infiltration and activation of T and B cells, macrophages, and mast cells [4,17].

There is evidence that the inflammatory process plays an important role in the development of degeneration and there is a significant correlation between the levels of inflammatory markers and the degree of degeneration [18-20]. Xue et al. have reported that as compared to healthy controls, patients with lumbar disc herniation (LDH) had significantly higher serum IL-21 and IL-17 levels. They also reported higher IL-21, IL-17, and cyclooxygenase-2 (COX-2) expression levels in disc tissues obtained from patients with LDH compared to normal disc tissues; visual analog scale (VAS) pain scores showed a positive correlation with serum IL-21 levels, and inflammation was responsible for LDH-related pain [21]. Likewise, a recent study showed that IL-21 played a role in the pathological development of intervertebral disc degeneration and it may worsen intervertebral disc degeneration by inducing TNF-α [18]. It is known that IL-21 plays an important role in the persistence and differentiation of both T and B cells [22]. Another study has reported an association between high-sensitivity CRP (hs-CRP) levels and severe pain (VAS of >40) [23].

There are a few studies in the literature that have examined the relationship between CDH and serum inflammatory markers. No study has yet investigated the relationship between NLR, PLR, and CRP levels in patients with neck pain accompanying cervical disc hernia but normal MR findings. Recently, Yılmaz et al. reported that NLR was an independent predictor in patients with lumbar disc hernia and low back pain [20]. Similarly, another study showed that preoperative and postoperative pain was more severe among patients with lumbar disc hernia and a higher NLR level, as an indicator of inflammation [24]. Dagistan et al. found no significant difference between serum NLR and PLR levels of the LDH patients and healthy controls [25]. In our study, the group with cervical disc hernia had a significantly higher NLR level as compared to the patients with neck pain but normal MRI and healthy controls.

In a recent study, it was reported that inflammatory cytokines (IL-6, IL-8) in disc samples obtained from humans were found to be significantly higher in multi-level compared to single-level [26]. In our study, there was no significant difference between single-level and multi-level CDH patients in terms of inflammatory parameters, while it was significantly higher in multi-level CDH patients than in the control group. As the compressed nerve tissue increases, it causes an increase in the inflammatory response, and these findings may make it easier to monitor if the damaged nerve tissue has increased. We believe that the reason for the lack of a significant difference between the CDH levels groups was probably the small sample size, and this subject should be further studied in larger patient groups.

Similarly, a recent study found that patients with extruded disc hernia had significantly higher mean serum hs-CRP levels compared to patients with bulging disc hernia and significantly higher mean serum IL-21 levels compared to patients with protruded disc hernia, which the authors potentially attributed to inflammation around nerve roots [19,27]. Sugimori et al. have found significantly higher mean hs-CRP levels among patients with LDH than the controls; they reported no significant correlation between hs-CRP levels and herniation level [28]. Our literature review showed that no study to date has specifically studied serum CRP levels in patients with cervical disc hernia. In our study, the mean serum CRP level was significantly higher in the CDH group than the control group, although we found no significant difference with respect to the CDH levels.

The limitations of our study include its relatively small sample size, retrospective design, and the lack of repeat tests to check if systemic inflammatory response subsided in patients with CDH.

## Conclusions

Higher NLR and CRP in patients with CDH compared to patients with neck pain but normal cervical MR and the controls may be due to a developing inflammatory response. The detection of significantly higher NLR in multi-level CDH patients compared to the control group is an indication that the inflammation increases as the affected nerve tissue increases. NLR and CRP are easy-to-use, rapid, and inexpensive indicators of systemic inflammation. It may be speculated that among patients with neck pain, those with non-elevated NLR levels may have normal neck MR imaging. Similarly, among patients with elevated NLR levels, early protective approaches may play a preventive role in disc degeneration and cervical disc hernia development. These findings warrant prospective studies involving larger patient groups.

## References

[1] Fejer R, Kyvik KO, Hartvigsen J (2006). The prevalence of neck pain in the world population: a systematic critical review of the literature. Eur Spine J.

[2] Peng B, DePalma MJ (2018). Cervical disc degeneration and neck pain. J Pain Res.

[3] Yin W, Bogduk N (2008). The nature of neck pain in a private pain clinic in the United States. Pain Med.

[4] Risbud MV, Shapiro IM (2014). Role of cytokines in intervertebral disc degeneration: pain and disc content. Nat Rev Rheumatol.

[5] Kang JD, Georgescu HI, McIntyre-Larkin L, Stefanovic-Racic M, Evans CH (1995). Herniated lumbar intervertebral discs spontaneously produce matrix metalloproteinases, nitric oxide, interleukin-6, and prostaglandin E2. Spine (Phila Pa 1976).

[6] Castell JV, Gómez-Lechón MJ, David M, Fabra R, Trullenque R, Heinrich PC (1990). Acute-phase response of human hepatocytes: regulation of acute-phase protein synthesis by interleukin-6. Hepatology.

[7] Aktürk S, Büyükavcı R (2017). Evaluation of blood neutrophil-lymphocyte ratio and platelet distribution width as inflammatory markers in patients with fibromyalgia. Clin Rheumatol.

[8] Li H, Zhou Y, Ma Y, Han S, Zhou L (2017). The prognostic value of the platelet-to-lymphocyte ratio in acute coronary syndrome: a systematic review and meta-analysis. Kardiol Pol.

[9] Imtiaz F, Shafique K, Mirza SS, Ayoob Z, Vart P, Rao S (2012). Neutrophil lymphocyte ratio as a measure of systemic inflammation in prevalent chronic diseases in Asian population. Int Arch Med.

[10] Templeton AJ, McNamara MG, Šeruga B (2014). Prognostic role of neutrophil-to-lymphocyte ratio in solid tumors: a systematic review and meta-analysis. J Natl Cancer Inst.

[11] Zheng SH, Huang JL, Chen M, Wang BL, Ou QS, Huang SY (2018). Diagnostic value of preoperative inflammatory markers in patients with glioma: a multicenter cohort study. J Neurosurg.

[12] Tao C, Wang J, Hu X, Ma J, Li H, You C (2017). Clinical value of neutrophil to lymphocyte and platelet to lymphocyte ratio after aneurysmal subarachnoid hemorrhage. Neurocrit Care.

[13] Auezova R, Ryskeldiev N, Doskaliyev A (2016). Association of preoperative levels of selected blood inflammatory markers with prognosis in gliomas. Onco Targets Ther.

[14] Brisby H, Olmarker K, Larsson K, Nutu M, Rydevik B (2002). Proinflammatory cytokines in cerebrospinal fluid and serum in patients with disc herniation and sciatica. Eur Spine J.

[15] Olmarker K, Rydevik B, Nordborg C (1993). Autologous nucleus pulposus induces neurophysiologic and histologic changes on porcine cauda equina nerve roots. Spine (Phila Pa 1976).

[16] Takahashi H, Suguro T, Okazima Y, Motegi M, Okada Y, Kakiuchi T (1996). Inflammatory cytokines in the herniated disc of the lumbar spine. Spine (Phila Pa 1976).

[17] Navone SE, Marfia G, Giannoni A (2017). Inflammatory mediators and signalling pathways controlling intervertebral disc degeneration. Histol Histopathol.

[18] Yılmaz A, Altaş H, Yıldırım T, Kaygısız Ş, Işık HS (2019). The clinical predictive value of the neutrophil to lymphocyte ratio as a biomarker in lumbar disc herniation. Middle Black Sea J Health Sci.

[19] Chen B, Liu Y, Zhang Y, Li J, Cheng K, Cheng L (2017). IL-21 is positively associated with intervertebral disc degeneration by interaction with TNF-α through the JAK-STAT signaling pathway. Inflammation.

[20] de Souza Grava AL, Ferrari LF, Defino HL (2012). Cytokine inhibition and time-related influence of inflammatory stimuli on the hyperalgesia induced by the nucleus pulposus. Eur Spine J.

[21] Xue H, Yao Y, Wang X (2015). Interleukin-21 is associated with the pathogenesis of lumbar disc herniation. Iran J Allergy Asthma Immunol.

[22] Zhu X, Ma D, Zhang J, Peng J, Qu X, Ji C, Hou M (2010). Elevated interleukin-21 correlated to Th17 and Th1 cells in patients with immune thrombocytopenia. J Clin Immunol.

[23] Stürmer T, Raum E, Buchner M, Gebhardt K, Schiltenwolf M, Richter W, Brenner H (2005). Pain and high sensitivity C reactive protein in patients with chronic low back pain and acute sciatic pain. Ann Rheum Dis.

[24] Bozkurt H, Arac D, Cigdem B (2019). Effect of preoperative uric acid level and neutrophil / lymphocyte ratio on preoperative and postoperative visual analogue pain scores in patients with lumbar disc herniation: a cross-sectional study. Turk Neurosurg.

[25] Dagistan Y, Dagistan E, Gezici AR, Halicioglu S, Akar S, Özkan N, Gulali A (2016). Could red cell distribution width and mean platelet volume be a predictor for lumbar disc hernias?. Ideggyogy Sz.

[26] Sadowska A, Touli E, Hitzl W, Greutert H, Ferguson SJ, Wuertz-Kozak K, Hausmann ON (2018). Inflammaging in cervical and lumbar degenerated intervertebral discs: analysis of proinflammatory cytokine and TRP channel expression. Eur Spine J.

[27] Talghini S, Vahedi A, Lotfinia I (2013). Discriminating extrusive and bulging disk herniations by using serum hs CRP. Pak J Biol Sci.

[28] Sugimori K, Kawaguchi Y, Morita M, Kitajima I, Kimura T (2003). High-sensitivity analysis of serum C-reactive protein in young patients with lumbar disc herniation. J Bone Joint Surg Br.

